# Berberine Improves Glucose Metabolism in Diabetic Rats by Inhibition of Hepatic Gluconeogenesis

**DOI:** 10.1371/journal.pone.0016556

**Published:** 2011-02-03

**Authors:** Xuan Xia, Jinhua Yan, Yunfeng Shen, Kuanxiao Tang, Jun Yin, Yanhua Zhang, Dongjie Yang, Hua Liang, Jianping Ye, Jianping Weng

**Affiliations:** 1 Department of Endocrinology, The Third Affiliated Hospital of Sun Yat-Sen University, Guangzhou, China; 2 Department of gastrointestinal-pancreatic surgery, The First-Affiliated Hospital, Sun Yat-Sen University, Guangzhou, China; 3 Pennington Biomedical Research Center, Louisiana State University, Eunice, Louisiana, United States of America; University of Hong Kong, China

## Abstract

Berberine (BBR) is a compound originally identified in a Chinese herbal medicine Huanglian (Coptis chinensis French). It improves glucose metabolism in type 2 diabetic patients. The mechanisms involve in activation of adenosine monophosphate activated protein kinase (AMPK) and improvement of insulin sensitivity. However, it is not clear if BBR reduces blood glucose through other mechanism. In this study, we addressed this issue by examining liver response to BBR in diabetic rats, in which hyperglycemia was induced in Sprague-Dawley rats by high fat diet. We observed that BBR decreased fasting glucose significantly. Gluconeogenic genes, Phosphoenolpyruvate carboxykinase (PEPCK) and Glucose-6-phosphatase (G6Pase), were decreased in liver by BBR. Hepatic steatosis was also reduced by BBR and expression of fatty acid synthase (FAS) was inhibited in liver. Activities of transcription factors including Forkhead transcription factor O1 (FoxO1), sterol regulatory element-binding protein 1c (SREBP1) and carbohydrate responsive element-binding protein (ChREBP) were decreased. Insulin signaling pathway was not altered in the liver. In cultured hepatocytes, BBR inhibited oxygen consumption and reduced intracellular adenosine triphosphate (ATP) level. The data suggest that BBR improves fasting blood glucose by direct inhibition of gluconeogenesis in liver. This activity is not dependent on insulin action. The gluconeogenic inhibition is likely a result of mitochondria inhibition by BBR. The observation supports that BBR improves glucose metabolism through an insulin-independent pathway.

## Introduction

Berberine (BBR, C_20_H_18_NO_4_) is an isoquinoline alkaloid originally isolated from the Chinese herb Coptis chinensis (Huanglian) [1]. BBR is widely used to reduce blood glucose in type 2 diabetes in China [1]. The mechanism is related to activation of AMPK (Adenosine monophosphate-activated protein kinase), ACC (acetyl-CoA carboxylase) and improvement of fatty acid oxidation [2]. BBR may improve insulin sensitivity through AMPK pathway or induction of insulin receptor expression [2], [3], [4]. We reported that AMPK activation by BBR is dependent on mitochondrial inhibition [5], which leads to an increase in the AMP/ATP(adenosine mono-phosphate/adenosine triphosphate) ratio in cells after BBR treatment. The AMP elevation is a result of the complex I inhibition in the mitochondrial respiratory chain [6]. The complex I inhibition blocks AMP conversion into ATP in the mitochondria. Therefore, BBR may reduce fasting blood glucose through an insulin-independent signaling pathway, such as AMPK activation and insulin receptor expression. This pathway is important in the skeletal muscle and adipose tissue in the insulin-induced glucose deposition. Hepatic gluconeogenesis contributes to elevation of fasting glucose, and requires mitochondrial production of ATP. We propose that the mitochondrial inhibition by BBR may contribute to the fasting glucose reduction through ATP depletion. In this study, we addressed this issue by studying hepatic gluconeogenesis in diabetic rats.

Hyperglycemia in type 2 diabetes is characterized by enhanced glucose production in the liver and kidney. Fasting blood glucose is determined by de novo glucose production (in liver and kidney) and glucose deposition in peripheral tissues. In the presence of insulin resistance, enhanced glucose output by liver contributes to hyperglycemia together with reduced glucose deposition in skeletal muscle, heart and adipose tissue. Inhibition of hepatic glucose production contributes to glycemic control in the diabetic patients by insulin sensitizers. Insulin inhibits expression of phosphoenolpyruvate carboxykinase (PEPCK) and glucose-6-phosphatase (G6Pase), two rate-limiting genes in gluconeogenesis. These genes are induced by glucagon and reduced by insulin. Activation and inhibition of transcription factors including forkhead transcription factor O1 (FoxO1), hepatic nuclear factor 4 (HNF4), and peroxisome proliferator-activated receptor-γ coactivator-1α (PGC-1α) are the underlying mechanism of the hormone actions [7], [8], [9]. The activities of these transcription factors are highly dependent on ATP supply. In the transcriptional process, transcriptional initiation, RNA elongation and RNA splicing all requires ATP supply, which provides energy to enzymes at each step. When ATP is reduced in cells, such as under hypoxia, gene transcription is suppressed in response to energy depletion [10]. Additionally, mRNA translation will be decreased leading to suppression of protein synthesis. The reduction in gene transcription and protein synthesis may lead to inhibition of hepatic gluconeogenesis. Given that BBR inhibits mitochondrial function, this possibility may represent a new mechanism for BBR in the control of blood glucose.

In the present study, we examined the molecular mechanism of hepatic gluconeogenesis in response to BBR in rats. We observed: (1) BBR decreased fasting blood glucose and expression of key gluconeogenic genes, such as PEPCK and G6Pase; (2) BBR suppressed the transcription factors, FoxO1 and sterol regulatory element-binding protein 1c (SREBP1); (3) BBR inhibited mitochondrial function in hepatocytes.

## Materials and Methods

### Type 2 diabetes rat model

The type 2 diabetic rat model was developed using male Sprague-Dawley rats (7–8 weeks, 200∼250 g) according to a protocol published elsewhere [11]. The rats were fed on high fat diet (63.47% calorie in fat) for 8 weeks, and then followed by a single low-dosage intraperitoneal injection of streptozotocin (STZ, 30 mg/kg, Sigma, St Louis, MO) after 12-hour fast. The non-diabetic control rats were fed on chow diet. Diabetic rats were divided into two subgroups: vehicle treated and BBR treated (n = 9). Non-diabetic rats were subgrouped in the same way. Plasma glucose increased in diabetic rats to 16.7 mmol/L within 3 days after STZ treatment and remained at this level throughout the experiment. These rats were used in the type 2 diabetic model. All procedures were performed in accordance with the principles of laboratory animal care (National Institutes of Health publication no. 85-23, revised 1985) and the animal protocol was approved by the Sun Yat-Sen University, Institutional Animal Care and Use Committee (IACUC, Approval ID:2008010102).

### Berberine treatment

The BBR intervention was initiated on the third day after STZ injection and conducted for five weeks. BBR solution was prepared in PBS and delivered by oral gavage at dosage of 380 mg·kg^−1^·d^−1^. The control group was given vehicle PBS.

### Blood and liver collection

After five weeks' BBR treatment, liver and blood were collected from each rat under anesthetized. Blood was collected from abdominal aorta using syringe puncture. Liver was removed and divided into three pieces, which were subjected to 4% formaldehyde fixation, Oil Red O staining and protein/RNA extraction. Tissue for protein/RNA preparation was stored in −80°C freezer after snap-frozen in liquid nitrogen. The sample collection was conducted in fasting (12-hour) and non-fasting conditions, respectively.

### Blood biochemistry

Fasting blood glucose was determined in the tail vein blood using portable glucometer (Roche, Basel, Switzerland). Blood insulin was measured in serum derived from the abdominal aorta blood with an insulin radioimmunoassay kit, which was purchased from Linco Research (Missouri, USA). Serum triglyceride and total cholesterol, alanine aminotransferase (ALT) and aspartate transaminase (AST) were determined using automatic biochemistry analyzer. Energy balance (diet intake, calorie intake, urine volume, and body weight) was monitored in individual rat for 3 consecutive days using a “metabolic cage”. The data were collected every day.

### Glucose tolerance and insulin tolerance tests

Oral glucose tolerance test (GTT) was performed 48 hours after the five-week BBR intervention. Glucose (2.0 g·kg^−1^ body weight) was administered through oral gavage after 12-hour fast. Blood glucose was determined at 0, 30, 60, 90 and 120 minutes after glucose challenge. Insulin tolerance test was carried out by intraperitoneal injection with insulin (0.75 U.kg^−1^ body wt). Blood glucose was checked at 0, 15, 30, 45, 60, and 90 minutes after insulin injection. Food intake, urine volume, diet intake were tested in each rat individually.

### Insulin releasing test

Insulin releasing test (IRT) was performed during the glucose tolerance test. Serum sample was collected at 0, 15, 30, 60, 90 and 120 minutes after glucose gavage. Serum insulin was determined by radioimmunoassay kit (Linco Research, Missouri, USA).

### Preparation for total, nuclear and cytoplasmic protein extracts

To determine insulin signaling activity, we collected the liver tissue at 30 minutes after intraperitoneal insulin injection (0.75 U.kg^−1^ body wt) and stored the samples at −80°C. The frozen liver sample was minced in ice cold phosphate buffered saline and homogenized in cell lysis buffer (Cell Signaling Technology Inc., Danvers, MA). The whole cell protein was collected after centrifugation at 4°C. The protein concentration was measured using the BCA protein assay kit (Sangon Biotech, Shanghai, China). The nuclear and cytoplasm protein were extracted from liver tissue using a Nuclear Extraction Kit (Chemicon, Inc, USA).

### Western blot

Protein (30 µg) was resolved in 10% sodium dodecyl sulfate polyacrylamide gel (SDS-PAGE) and transferred to a PVDF membrane. The antibodies to Akt (Protein kinase B, PKB), PEPCK, G6Pase, fatty acid synthase (FAS), FoxO1, SREBP1, SREBP2, carbohydrate responsive element-binding protein (ChREBP), TFIID (the TATA box binding protein, TBP) were purchased from the Santa Cruz Biotechnology (CA, USA). Antibodies to glyceraldehyde-3-phosphate dehydrogenase (GADPH) and β-actin were from the Cell Signaling Technology, USA. The peroxide-conjugated anti-rabbit antibody (Boster, Wuhan, China) was used to detect the protein signal with enhanced chemiluminescence system (Pierce, USA). The band intensity was quantified by densitometry (Calibrate densitometer, Bio-Rad, Inc).

### RNA isolation and quantitative real-time reverse transcriptase polymerase chain reaction (qRT-PCR)

Total RNA was extracted from the frozen liver tissues using the TRIzol RNA isolation reagent (Invitrogen, Carlsbad, CA). Reverse transcription of 1 µg RNA was carried out according to the instructions of Prime Script™ 1st Strand cDNA Synthesis Kit (TaKaRa, Japan). The qRT-PCR reaction was conducted in 20 µl (SYBR® Premix Ex TaqTM, TaKaRa Japan). The result was normalized against β-actin mRNA signal. Primer sequences were as follows: Rat PEPCK forward primer (5′-CTCACCTCTGGCCAAGATTGGTA-3′) and reverse primer (5′-GTTGCAGGCCCAGTTGTTGA-3′); Rat G6Pase forward primer (5′-AACGTCTGTCTGTCCCGGATCTAC-3′) and reverse primer (5′-ACCTCTGGAGGCTGGCATTG-3′); Rat FAS forward primer (5′-CACAGCATT CAGTCCTATCCACAGA-3′) and reverse primer (5′-CACAGCCAACCAGATGCTTCA-3′); Rat FoxO1 forward primer (5′-TGCCAAACTCACTACACCACTT-3′) and reverse primer (5′-ACGATCAGGTTCCGTCATTC-3′); Rat β-actin forward primer (5′-GGAGATTACTGCCCTGGCTCCTA-3′) and reverse primer (5′-GACTCA TCGTACTCCTGCTTGCTG-3′).

### Histology

The liver sample fixed in 4% Para formaldehyde was paraffin-embedded, sectioned at 8 µm in thickness and stained with hematoxylin & eosin dye (HE) and immunohistochemistry (DAB staining). The frozen samples were sectioned and stained with Oil Red O and Sudan III. The immunohistostaining was performed according to instruction of the SABC assay (Boster, Wuhan, China). Paraffin-embedded sections were deparaffinized using xylene and re-hydrated in a series of alcohols, and incubated in antigen retrieval (0.01 M citrate buffered saline, pH 6.0) for 5 min at 95°C and then for 20 min at room temperature. The sections were then treated with 3% hydrogen peroxide for 5 min. The slide was treated with 10% normal goat serum to prevent nonspecific antibody binding. The sample was treated with the primary antibody overnight at 4°C in PBS. Imaging was obtained using a Nikon microscope and analyzed by software (HMIAS-2000, Championimage Inc, China).

### Oxygen consumption and AMP/ATP ratio

Oxygen consumption and AMP/ATP ratio were examined in H4IIE hepatocytes using protocols reported elsewhere [5]. The cells were cultured in a plate embedded with an oxygen-sensitive dye. After 6 hrs, BBR was added to the medium to treat cells for 12 hrs. The oxygen consumption was determined by the fluorescence intensity in the oxygen-sensitive dye, and expressed as relative fluorescence units (NRFU). The reading was normalized with the background in a blank well. The cells were treated with 20 µM BBR for 16 hrs in serum-free medium containing 0.25% BSA. The cells were lyzed in 0.6 N HClO_4_ and neutralized with 1 N KHCO_3_. Then AMP and ATP contents were measured using high-performance liquid chromatography (HPLC).

### Statistical analysis

The results were expressed as the means ± SE. The significant difference in two groups was statistically analyzed using the Student's t test. The significant difference in more than two groups was statistically analyzed using ANOVA. The least-square deconvolution (LSD) was performed to detect differences between two groups. Statistical significance was set at *P*<0.05 (two tails).

## Results

### Characteristics of the experimental animals treated by BBR

To investigate BBR action in liver, we used diabetic rats, which were generated after 8 weeks high fat diet feeding and a single streptozotocin treatment. The rats were treated with BBR through oral gavage for 5 weeks, and then subjected to variety of tests. BBR reduced body weight, fasting blood glucose, total cholesterol, and triglycerides in the diabetic rats (Table 1). Calorie intake, serum insulin, urine volume, water intake, serum ALT, and AST were not altered by BBR. These data suggest that the reduction in blood glucose and lipids may be a result of enhanced energy expenditure in the rats. These were observed with reduced body weight in the absence of food intake reduction. The body weight was also decreased in the non-diabetic rats (lean rats) by BBR (Table 1), suggesting that BBR reduces body weight in both obese and lean rats.

**Table 1:** Characteristics of the experimental animals at the end of intervention study (n = 9).

Index	Diab+BBR	Diab	Nor	Nor+BBR
Body weight (g)	462±34.6 a b	495±29.6	489±34.2	452±21.8 a b
Water intake (ml·g^−1^·d^−1^)	0.2749±0.0530 b	0.3495±0.0141 b	0.1272±0.0129 a	0.1272±0.0129 a
Urine volume (ml·g^−1^·d^−1^)	0.2569±0.0281 b	0.2432±0.0583 b	0.0339±0.0071 a	0.0339±0.0071 a
Diet intake (g·g^−1^·d^−1^)	0.067±0.004 a	0.067±0.014 b	0.067±0.005	0.070±0.006
Calorie intake (kcal·g^−1^·d^−1^)	0.383±0.02 b	0.385±0.07 b	0.227±0.02 a	0.236±0.03 a
FBG (mmol/L)	7.30±1.19 a b	20.63±1.45 b	4.10±0.17 a	4.30±0.36 a
FINS (ng/mL)	0.136±0.009	0.141±0.007	0.121±0.001	0.132±0.002
TC (mmol/L)	1.03±0.11 a,b	2.41±0.45 b	1.55±0.16 a	1.59±0.57 a
TG (mmol/L)	1.52±0.10 a	4.54±5.31 b	1.28±0.99 a	1.36±0.33 a
ALT (U/L)	69.60±18.73	84.43±31.14 b	65.50±24.61 a	59.67±19.63 a
AST (U/L)	208.38±22.94 b	245.50±43.77 b	138.85±26.30 a	172.37±16.35 a

BBR, berberine; Diab+BBR, diabetic rats treated with berberine; Nor, normal lean rats; Nor+BBR, normal rats treated with berberine; FBG, fasting blood glucose; FINS, fasting serum insulin; TC, total cholesterol; TG, triglycerides; ALT, alanine aminotransferase; AST, aspartate transaminase. Data are presented as mean ± SE (n = 9).

a : *P*<0.05, compared with diabetic rats;

b : *P*<0.05, compared with normal rats(Nor).

**Figure 1:**
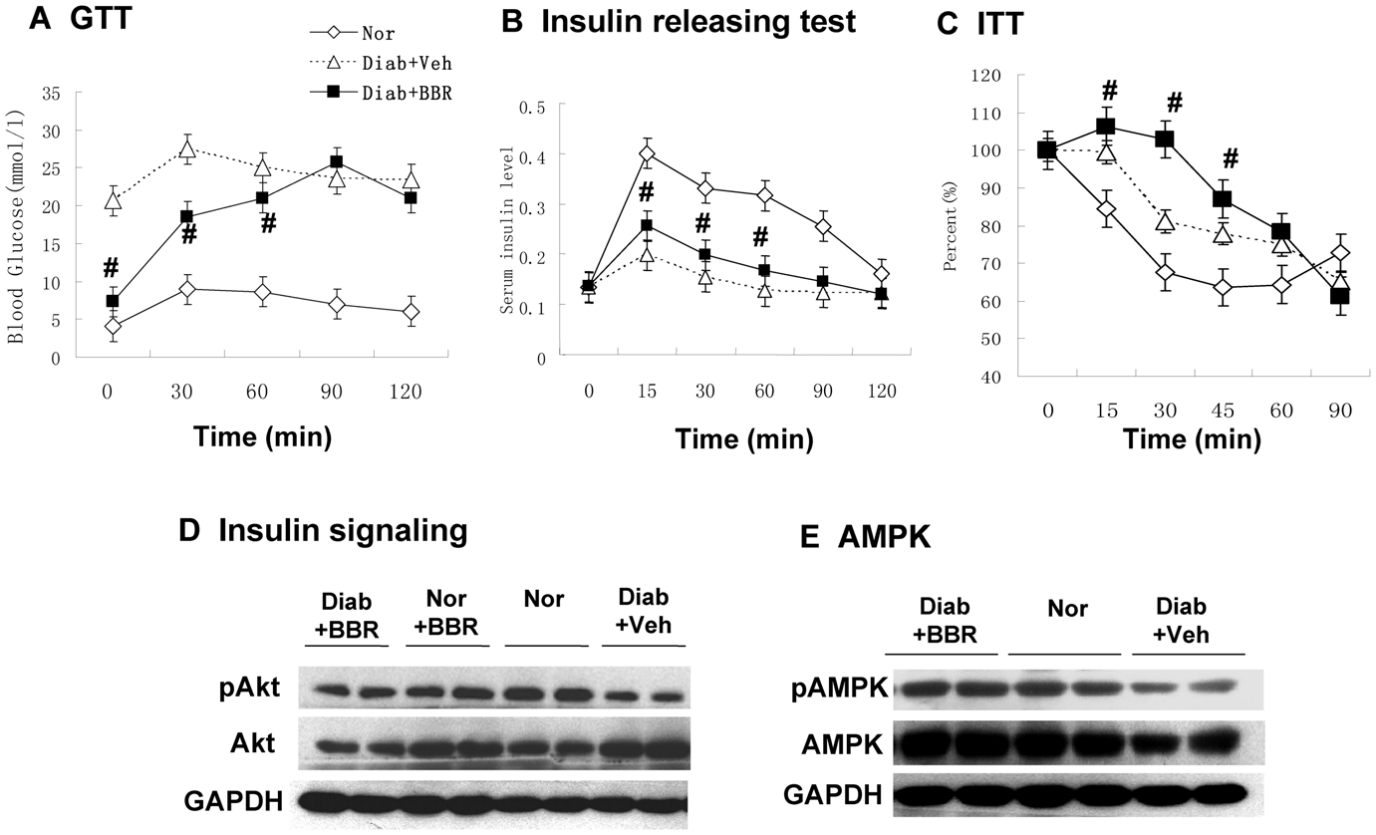
BBR decreased fasting blood glucose without improvement in insulin sensitivity. (A) Effects of BBR treatment on OGTT in diabetic rats. Oral glucose tolerance test (OGTT) was conducted with glucose 2 g.kg^−1^ body wt after 5 week BBR treatment (380 mg.kg^−1^ day^−1^) (n = 6). (B) Insulin release during the OGTT (n = 6). (C) ITT test (n = 6). The test was conducted after 8 hour fasting with insulin (0.75 U.kg^−1^ body wt). Nor, normal rats without obesity; Diab+Veh: Diabetic rats treated with vehicle; Diab+BBR, diabetic rats treated with BBR. # *P*<0.05, compared with Diab+Veh. (D) Insulin signaling. Phosphorylation of Akt (Ser473). Rats were challenged with insulin (0.75 U.kg^−1^, intraperitoneal injection) and liver was collected in 30 minutes. The Akt assay was performed in a Western blot. Loading control is GAPDH. (E) Expression and phosphorylation of AMPK (Thr172). # *P*<0.05, compared with Diab+Veh group. * *P*<0.05, compared with normal group (Nor).

Systemic insulin sensitivity was evaluated by fasting glucose, GTT and ITT. In the GTT test, the BBR group exhibited lower blood glucose than the un-treated control at the basal level (Fig. 1A). After oral glucose challenge, the untreated group had little increase in blood glucose. The blood glucose only increased modestly by the glucose challenge. However, the BBR group exhibited a dramatic increase in blood glucose at 30 and 60 minutes. The glucose reached to the same level as the untreated group at 90 and 120 minutes. In the test, blood insulin was monitored at each time point to determine the pancreatic beta cell function. The BBR group and untreated group had identical basal insulin. However, the BBR group exhibited a significantly higher insulin level than the untreated group between 15–60 minutes after the glucose challenge (Fig. 1B). In the ITT test, both the BBR group and untreated group exhibited similar change in percentage of blood glucose at 60–90 minutes, but the BBR group had less response than the untreated group during 15–45 minutes (Fig. 1C), suggesting a delayed response to insulin in the BBR group. Those data demonstrate that BBR reduces fasting blood glucose without altering blood insulin in the basal condition, suggesting an increase in systemic insulin sensitivity by HOMA-IR. Though GTT and ITT results do not support that systemic insulin sensitivity was improved by BBR, the results may not be definitive. Hyperinsulinemic-euglycemic clamping is required to determine insulin sensitivity.

To test insulin action in liver, we examined the insulin signaling activity by checking Akt phosphorylation status in response to insulin. Liver was collected from rats in 30 minutes after insulin challenge. Akt phosphorylation status was determined using a phospho-specific antibody in a Western blot. The phosphorylation was not significantly improved in the BBR group (Fig. 1D), suggesting that BBR treatment does not improve insulin signaling in liver. We also examined AMPK in the liver. AMPK protein and its phosphorylation were both reduced in the diabetic rats (Fig. 1E). In the BBR group, the reduction was not observed, suggesting that BBR may protect the AMPK activity. AMPK improves glucose uptake by stimulating GLUT4 translocation in adipocytes and muscle cells. In hepatocytes, this mechanism is not working since hepatocytes do not express GLUT4. The data suggest that BBR does not alter insulin signaling pathway in liver.

### Berberine inhibits the expression of gluconeogenic genes in liver

**Figure 2:**
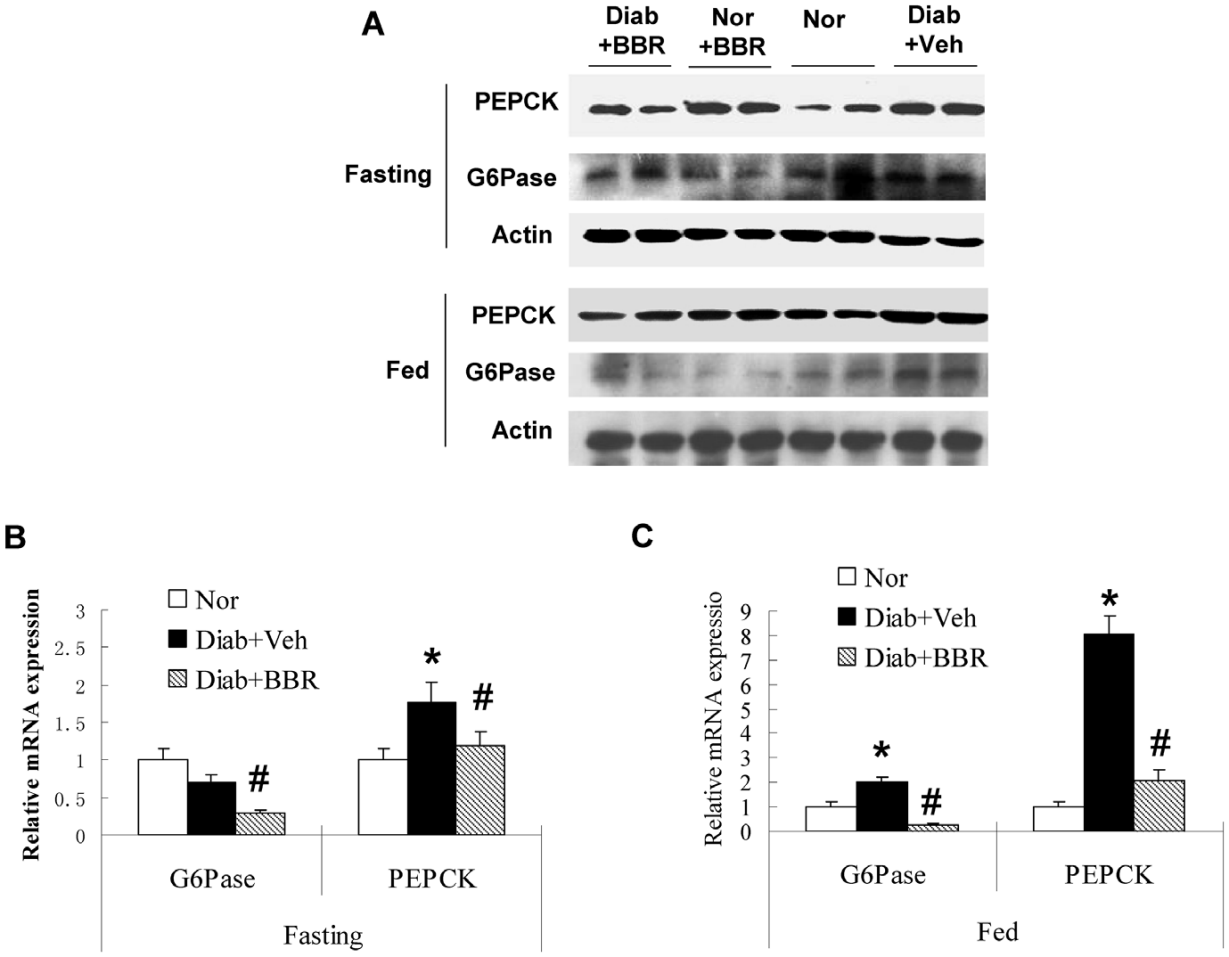
Expression of gluconeogenic genes in liver. (A) Protein levels of PEPCK and G6Pase. Total protein was made from liver tissue and used in a Western blot. (B) mRNA in fasting condition. Total RNA was extracted in from liver tissue and used in qRT-PCR. The signal was normalized with actin mRNA. (C) mRNA in fed condition. The mRNA data are presented as mean ± SEM (n = 6). # *P*<0.05, compared with Diab+Veh group. * *P*<0.05, compared with normal group (Nor).

To investigate hepatic gluconeogenesis, we examined expression of PEPCK and G6Pase in liver. PEPCK and G6Pase are two rate-limiting enzymes in the gluconeogenic process in hepatocytes. The protein levels of PEPCK and G6Pase are primarily controlled by gene transcription. In the liver of BBR group, both enzymes were reduced significantly in proteins (Fig. 2A). The changes were observed in both fasting and fed conditions. mRNA of PEPCK and G6Pase was also decreased in the BBR group, suggesting that protein reduction is a result of mRNA reduction (Fig. 2B and 2C). The inhibition was observed in the fasting condition, when insulin level is low in the blood (Table 1). Although insulin inhibits the two enzyme expression, the data do not support a role of insulin. The data suggest that BBR suppresses PEPCK and G6Pase expression independently of insulin.

### Berberine inhibits FoxO1 in liver

**Figure 3:**
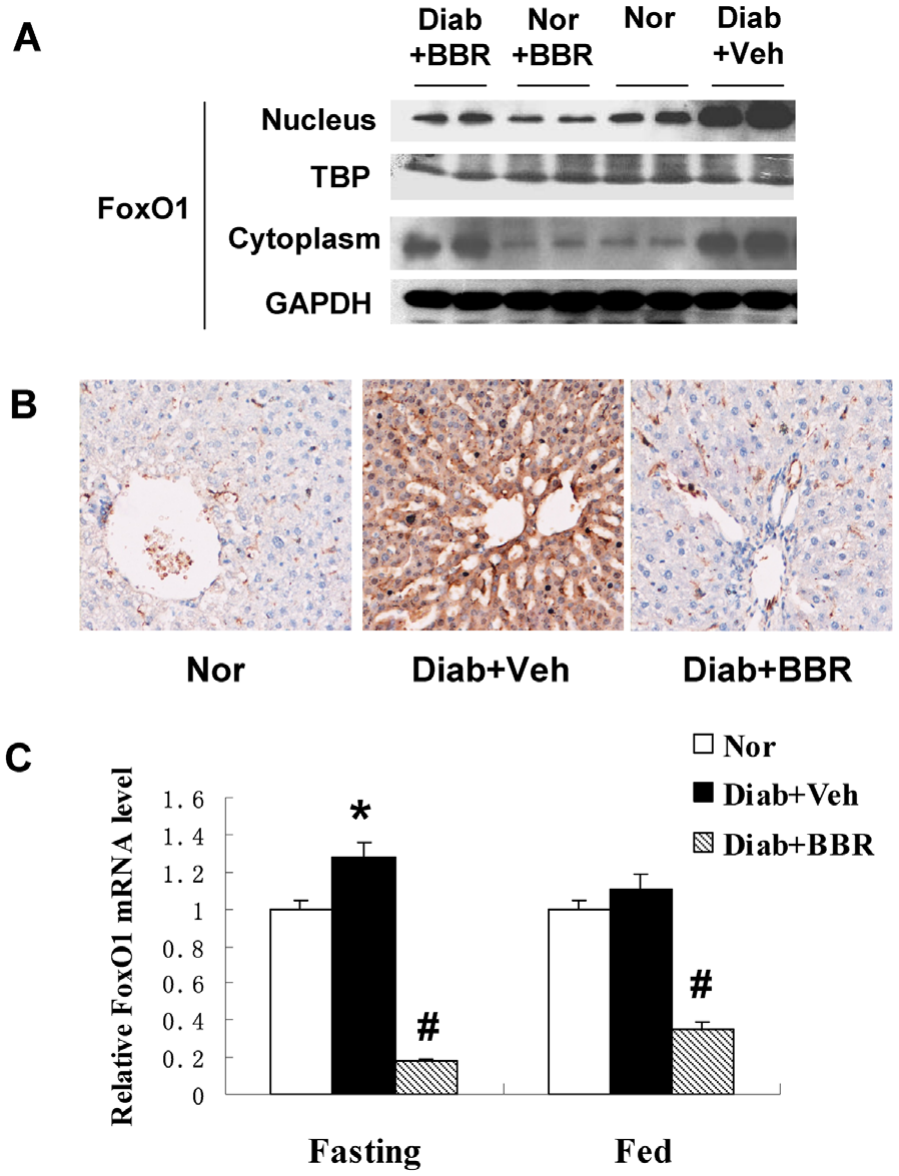
FoxO1 expression in liver. (A) FoxO1 protein in liver. Nuclear and cytoplasmic proteins were extracted from liver and analyzed in a Western blot. TBP (TATA-binding protein) and GAPDH proteins are loading controls in the nuclear and cytoplasmic proteins. (B) Immunohistostaining of FoxO1 protein in liver. DAB dye (brown) was used to indicate the FoxO1 protein signal; (C) mRNA expression of FoxO1. mRNA was quantified in real time RT-PCR (n = 6). # *P*<0.05, compared with Diab+Veh group; * *P*<0.05, compared with normal group (Nor).

FoxO1 is an important transcription factor in the control of gluconeogenesis in liver [9]. FoxO1 induces PEPCK and G6Pase gene transcription to initiate gluconeogenesis. This activity is dominant in fasting condition in the absence of a high level of insulin, which inactivates the transcriptional activity of FoxO1 through Akt-mediated nucleus exclusion and protein degradation of FoxO1. To understand the mechanism of PEPCK and G6Pase inhibition by BBR, we examined FoxO1 protein and mRNA in the liver. FoxO1 protein was higher in the liver of diabetic rats relative to the non-diabetic control (Fig. 3A). The elevation may be a result of reduced insulin activity from the low levels of insulin and insulin sensitivity in the diabetic rats. The FoxO1 protein was reduced by BBR. The reduction was observed in both cytoplasm and nucleus. However, the nuclear reduction was much stronger (Fig. 3A). The protein reduction was also observed in the liver tissue by immunohistostaining (DAB dye) (Fig. 3B). FoxO1 mRNA was reduced by BBR, and the inhibition degree was identical in both fasting and fed conditions (Fig. 3C). Given that insulin is low in the fasting condition, the data suggest that BBR inhibits FoxO1 expression independently of insulin.

### Berberine inhibits lipid accumulation in liver

**Figure 4:**
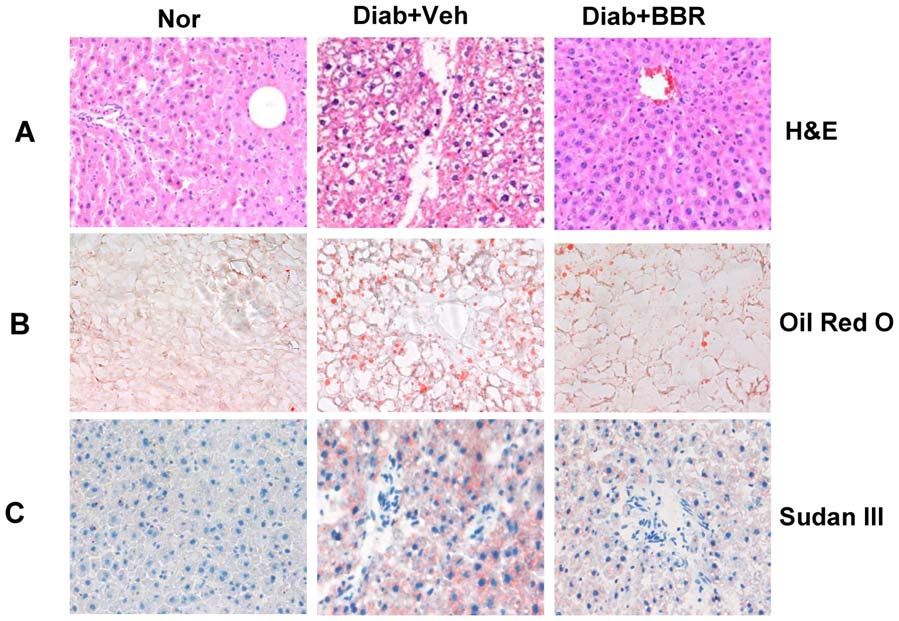
Liver steatosis in histology. (A) Hematoxylin and eosin (H & E) staining. (B) Oil Red O staining. (C) Sudan III staining. Pictures were taken under a microscopy with ×20 object lenses.

Lipid accumulation in liver impairs hepatocyte function leading to hyperglycemia through hepatic glucose production. We examined BBR impact on fat content in liver in the BBR-treated rats. Liver tissue was analyzed under microscope after HE staining and Oil Red O staining. Lipid accumulation was elevated in the diabetic rats as indicated by the unstained area in hepatocytes under HE staining (Fig.4A), and red color in hepatocytes under the Oil Red O or Sudan III staining (Fig. 4, B and C). The hepatocyte density and proliferation status were determined with DNA staining in blue color by hematoxylin (Fig. 4, A and C). Hepatocyte proliferation was enhanced in the diabetic condition as indicated by the loosen chromatin DNA in the nucleus of hepatocytes in the diabetic group (Fig. 4, A and C). The unpacking process is required for DNA duplication and chromosome doubling. In the BBR group, the change in DNA was inhibited, and DNA was packed tightly in the nucleus, suggesting a reduction in cell proliferation. The cell density was not altered by BBR. The data suggest that BBR inhibits lipid accumulation in hepatocytes. This is not a result of liver toxicity since BBR did not reduce cell density in liver. BBR may inhibit the metabolic activity in hepatocytes as cell proliferation activity is reduced by BBR.

### Berberine ameliorates fatty acid synthesis in liver by inhibiting expression of SREBP1 and FAS

**Figure 5:**
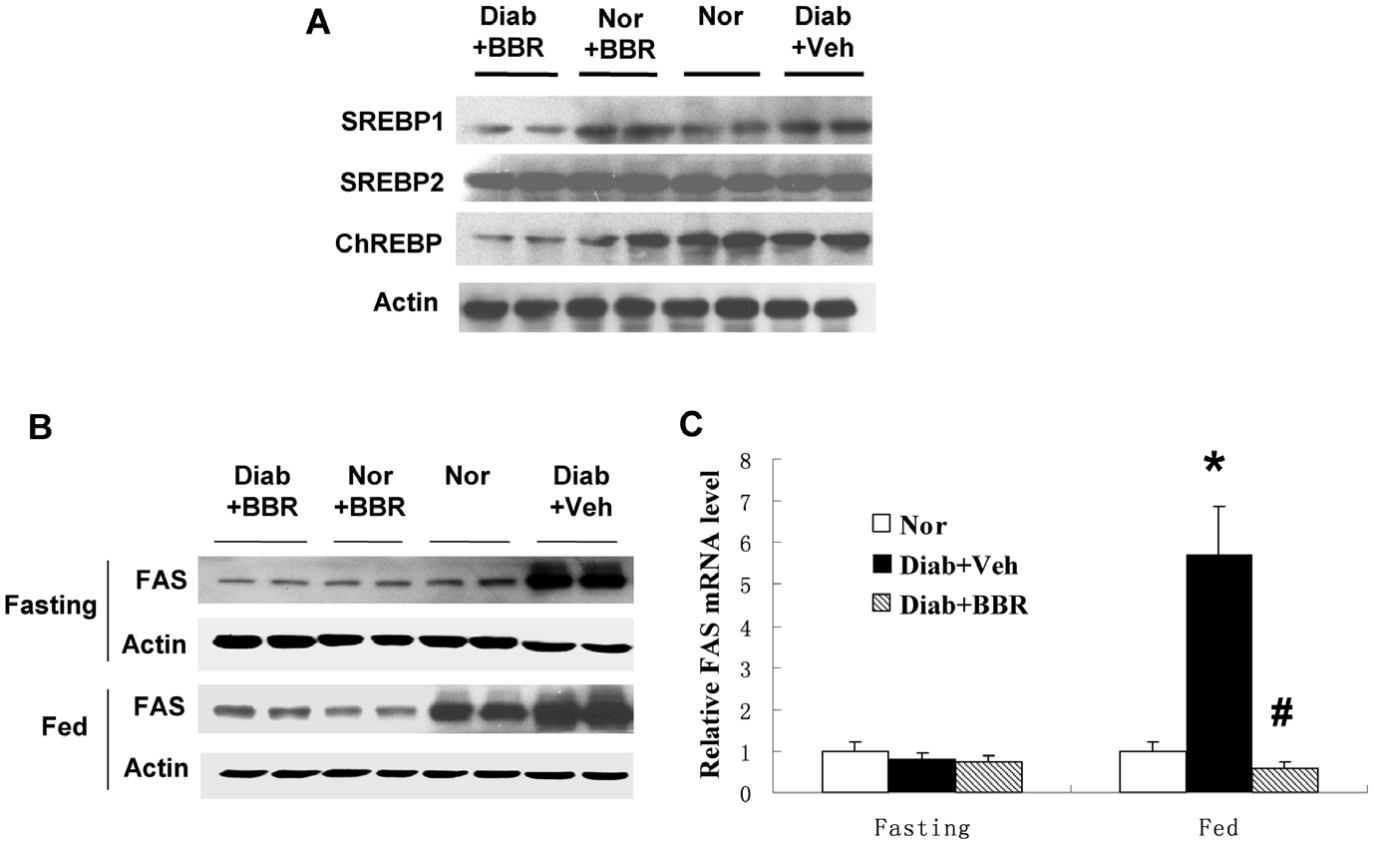
Lipogenesis in liver. (A) Lipogenic transcription factor proteins. Liver total protein was used in the Western blot. SREBP1, SREBP2 and ChREBP were detected in the fasted liver with specific antibodies. Beta-actin is a loading control. (B) FAS protein. The protein was detected in a Western blot in the fasted and fed liver, respectively. (C) FAS mRNA. mRNA was detected by qRT-PCR and normalized with beta-actin mRNA (n = 6). # *P*<0.05, compared with Diab+Veh group; * *P*<0.05, compared with normal group (Nor).

SREBP1 and ChREBP are transcription factors for lipogenic gene expression, such as FAS [12], [13]. Inhibition of SREBP1 by gene knockout was reported to decrease lipid accumulation in liver [14]. SREBP2 is an isoform of SREBP1 and has an activity in induction of cholesterol synthetic genes. It was reported that BBR reduces SREBP2 expression in hepatocytes [15]. To understand the molecular basis of lipid reduction in liver, we measured expression of SREBP1, SREBP2, ChREBP and FAS. Three genes were reduced in liver by BBR (Fig. 5). The SREBP1 total protein was determined in the liver tissue lysate and was decreased by BBR (Fig. 5A). A reduction was also observed in ChREBP. SREBP2 was not changed in the same condition. FAS expression was increased in liver under the fed condition (Fig. 5, B and C). The increase was observed even in the non-diabetic rats. However, in the diabetic rats, the increase was enhanced much more, suggesting a more active fatty acid synthesis in response to feeding. In response to the BBR treatment, the FAS increase was attenuated (Fig. 5, B and C). The reduction was observed in the diabetic rats as well as in the non-diabetic mice. These data suggest that BBR inhibits FAS through suppression of SREBP1 and ChREBP expression. The inhibition represents a mechanism of BBR prevention of fatty liver.

### Inhibition of mitochondrial function in hepatocytes by BBR

**Figure 6:**
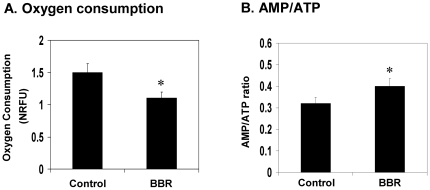
Inhibition of mitochondrial function by BBR in hepatocytes. (A) BBR decreased oxygen consumption in H4IIE hepatocytes. The cells were plated in DMEM culture medium supplemented with 10% FBS. Oxygen consumption was determined for 12 hrs after BBR (20 µM) treatment. (B) AMP/ATP ratio in H4IIE cells. The cells were treated with BBR in serum-free medium for 16 hours. AMP and ATP levels were determined using HPLC. The data are presented as mean ± SEM (n = 5). * *P*<0.05.

Mitochondria are the major subcellular organs in oxygen consumption. In mitochondria, oxygen is used to oxidize glucose and fatty acids in the production of ATP. The side products are water and carbon dioxide in mitochondria. Although ATP is also made in glycolysis, the efficiency of ATP production is much lower in this mitochondria-independent pathway. Glycolysis becomes dominant when mitochondrial function is inhibited for ATP production. Mitochondrial inhibition leads to reduction in oxygen consumption and an increase in the AMP/ATP ratio. Therefore, oxygen consumption and AMP/ATP ratio are two major indicators of mitochondrial function. In an early study, we reported that BBR inhibited mitochondrial function in adipocytes and myotubes [5]. The effect was not tested in hepatocytes. To test the role of mitochondrial inhibition in liver, we examined mitochondrial function in hepatocytes after BBR treatment in cell culture. BBR decreased oxygen consumption and increased AMP/ATP ratio in H4IIE cells, a rat hepatoma cell line (Fig. 6, A and B). The data suggest that the BBR suppresses mitochondrial function in hepatocytes. This activity may account for the reduced gene expression for gluconeogenesis and fatty acid synthesis in liver.

## Discussion

Our data suggest that BBR inhibits glucose production in liver. The inhibition is related to suppression of PEPCK and G6Pase expression. This is a new mechanism for BBR action in the regulation of glucose metabolism. In literature, BBR is reported to enhance insulin sensitivity in adipocytes and muscle cells. Activation of the AMPK pathway is a mechanism of enhanced glucose uptake in skeletal muscle [2], [5]. However, the role of AMPK pathway is controversial for the metabolic activities of BBR. The AMPK activity was reported in muscle (L6) and hepatocytes (HepG2) [16], [17], but the activity was not observed in 3T3-L1 adipocytes [18]. In HepG2 cells, AMPK was reported to mediate BBR activity in the inhibition of cholesterol and triglyceride synthesis [17]. However, AMPK was not tested in BBR action in the regulation of hepatic gluconeogenesis. In the current study, we addressed this issue by examining AMPK and gluconeogenesis in liver. We observed that BBR improved AMPK activity and inhibited gluconeogenic gene expression at the same time. In the diabetic rats, BBR treatment restored AMPK activity to the level of non-diabetic rats. The change in AMPK activity is associated with a reduction in PEPCK and G6Pase expression. AMPK activation is proposed as a result of mitochondria inhibition by BBR [5], [6]. Here, our data suggest that mitochondria inhibition is also a mechanism for suppression of hepatic gluconeogenesis and lipogenesis. The inhibition leads to suppression of gene expression and catalytic inactivation of related enzymes in liver through depletion of ATP. Recent literature supports that BBR increases glucagon-like peptide-1 (GLP-1) level in vivo and in vitro [19], [20]. This activity of BBR may contribute to AMPK activation by BBR as well.

We demonstrated that insulin signaling was not significantly modified by BBR in liver. BBR was reported to enhance glucose uptake through GLUT4 translocation in skeletal muscle [16], [21]. However, this mechanism is controversial as the GLUT4 activity was not confirmed in other studies [5], [18]. Our data suggest that even this AMPK-GLUT4 interaction is a mechanism for BBR action in vivo, its contribution to glucose metabolism in whole body is limited. Our data do not support the mechanism in vivo as the insulin sensitivity was not significantly improved by BBR in the diabetic rats. This conclusion is supported by a recent study, in which BBR was shown to cause muscle atrophy through induction of protein degradation [22]. This report argues against the BBR activity in insulin sensitization in muscle since insulin is known to prevent muscle atrophy [23], [24]. In the current study, we examined the insulin signaling pathway in liver by determining Akt phosphorylation status. We did not observe a significant change in the insulin signaling pathway following the BBR treatment. The current study suggests that BBR may regulate glucose metabolism independently of insulin signaling pathway in liver.

Inhibition of mitochondria ATP production may contribute to the suppression of hepatic gluconeogenesis and hepatic steatosis. We observed that oxygen consumption was reduced and the AMP/ATP ratio was increased by BBR in hepatocytes. The data provide evidence for this new action of BBR in mitochondria suppression in hepatocytes. This observation is consistent with BBR activity in the purified mitochondria, in which BBR inhibited NAD-linked respiration in mitochondria [25], [26], [27]. In hepatocytes, we observed that BBR enhanced lactate release [5], a sign of the mitochondria inhibition. Here, we demonstrated that oxygen consumption was reduced in hepatocytes by BBR, and this reduction was associated with an increase in AMP/ATP ratio. Though ATP production pathway is switched from mitochondrial oxidative phosphorylation to glycolysis in the presence of BBR, the glycolysis-generated ATP is not sufficient to maintain the intracellular ATP level in hepatocytes. The ATP depletion should account for inhibition of PEPCK and G6Pase expression through inactivation of transcription factors, whose activities are dependent on ATP.

The mitochondrial inhibition is a mechanism for inhibition of lipid accumulation in liver. We observed that BBR reduced hepatic steatosis in the diabetic rats. The mechanism is likely inhibition of lipogenesis in liver. BBR was shown to reduce plasma lipid and reduce plasma hepatic enzymes (ALT, AST, GGT) [28], [29]. This BBR activity was confirmed in the current study. In term of mechanism, BBR is reported to inhibit cholesterol synthesis and induce LDLR expression [17], [26]. In the current study, we observed that BBR inhibited fatty acid synthesis in liver. BBR inhibited FAS gene expression. Expression of lipogenic transcription factors (SREBP and ChREBP) was also reduced, which provides a mechanism for the FAS inhibition. Given that ATP is required for expression and function of the lipogenic transcription factors, the ATP depletion provides an answer to the lipogenic inhibition by BBR. The lipogenic inhibition may contribute to body weight loss in the BBR-treated rats observed in this study. BBR did not reduce food intake in our study. It has been reported that BBR does not affect expression of the appetite-regulating neuropeptides (POMC and NPY) in the hypothalamus in rodents [2]. Therefore, the reduction in hepatic steatosis is not a consequence of alteration in calorie intake.

Pharmacokinetics of BBR is different among tissues [30]. This character explains the long lasting AMPK activation that is observed in the current study. Recent studies suggest that: 1) BBR has a long half life in the liver. The BBR clearance rate is quite different among tissues, such as hippocampus VS plasma [31]. According to a recent report, there is a 70-fold difference in the BBR clearance rate in liver VS plasma in rats [32]. The studies suggest that BBR may have a longer half life in liver for the AMPK activation; 2) GLP-1 may mediate BBR's long-standing effect. BBR increases L cell activity in expression of GLP-1 in vivo [33], [34]. The improved L cell activity may remain in the absence of BBR for the elevated GLP-1 expression. If this is the case, GLP-1 will mediate BBR activity in the AMPK activation in liver after BBR withdrawn. In the future, we will test this possibility by examining GLP-1 level in our models.

**Figure 7:**
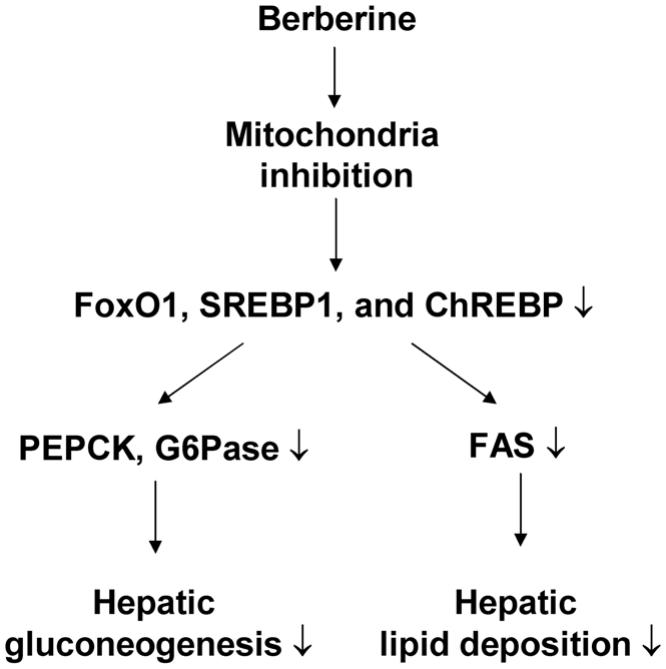
Schematic model of BBR signaling pathway. BBR inhibits mitochondria function and decrease intracellular ATP. This leads to a reduction in gluconeogenic and lipogenic transcription factors (FoxO1, SREBP1, and ChREBP). As a result, expression of gluconeogenic genes (PEPCK and G6Pase) and lipogenic gene (FAS) are decreased. These molecular changes represent a signaling pathway for improvement of fasting glucose and liver steatosis in the BBR-treated diabetic rats.

In summary, we conclude here that BBR reduces fasting blood glucose, attenuates gluconeogenesis and hepatic steatosis in type 2 diabetic rats (Fig. 7). Suppression of hepatic gluconeogenesis provides an excellent mechanism for the reduction in blood glucose. Inhibition of PEPCK and G6Pase expression is associated with decreased expression of transcription factors (FoxO1, SREBP1 and ChREBP) in the liver. BBR suppresses mitochondria function in hepatocytes to reduce ATP level. ATP depletion is a potential mechanism for BBR action in the inhibition of gluconeogenesis and lipogenesis in liver. The study suggests that inhibition of mitochondrial function in liver is responsible for the metabolic activities of BBR.
